# Ozonized Water-Mediated Maillard Reaction of Fructose-Glycine: Characterization and Antioxidant Properties

**DOI:** 10.3390/foods15020303

**Published:** 2026-01-14

**Authors:** Worawan Panpipat, Natthawadee Khaochamnan, Sutasinee Thongkhaow, Visaka Anantawat, Nisa Saelee, Roberto Castro-Muñoz, Manat Chaijan

**Affiliations:** 1 Food Technology and Innovation Research Center of Excellence, Division of Food Science and Innovation, Department of Food Industry, School of Agricultural Technology and Food Industry, Walailak University, Nakhon Si Thammarat 80161, Thailand; pworawan@wu.ac.th (W.P.); natthawadee680@gmail.com (N.K.); sutasineethongkhaow@gmail.com (S.T.); pvisaka@wu.ac.th (V.A.); snisa@wu.ac.th (N.S.); 2 Department of Sanitary Engineering, Faculty of Civil and Environmental Engineering, Gdansk University of Technology, G. Narutowicza St. 11/12, 80-233 Gdansk, Poland; roberto.castro-munoz@pg.edu.pl; 3 Institute of Sustainable Processes, University of Valladolid, Dr. Mergelina s/n., 47011 Valladolid, Spain

**Keywords:** maillard reaction products, antioxidant, ozone, emulsion, oxidation

## Abstract

This study investigates the use of ozonized water as a novel reaction medium for generating Maillard reaction products (MRPs) from fructose and glycine, comparing their physicochemical properties and antioxidant performance with those produced in phosphate buffer. Heating in ozonized water delayed early Maillard stages, as indicated by slower browning, lower A294 and A420 absorbance, and higher L* values. However, prolonged heating led to intensified reddish-brown coloration and elevated intermediate formation, suggesting ozone-modified reaction pathways. pH declined more sharply in the ozone system, while conductivity increased significantly after 60 min, reflecting accelerated late-stage reactions. Antioxidant activity, assessed via DPPH and ABTS assays, developed more slowly in the ozone system but reached comparable levels to the buffer after 120 min. In emulsion models, MRPs from either system alone exhibited pro-oxidant effects, while blends, especially those produced using ozonized water and buffer at ratios of 75:25 and 50:50, significantly enhanced oxidative stability. Zeta-potential analysis showed that emulsions containing MRP blends had less negative initial charges but exhibited greater stability over 3 days compared to those with individual treatments. These findings highlight the potential of ozonized water to modulate Maillard reaction kinetics and suggest that blending MRPs from different reaction media can enhance antioxidant functionality and emulsion stability in complex food systems.

## 1. Introduction

The Maillard reaction is a non-enzymatic browning process that occurs between amino and carbonyl groups. While it is well known for generating desirable flavors and aromas in cooked foods, its importance also lies in the formation of compounds with potential health and functional benefits. In particular, Maillard reaction products (MRPs) have gained increasing attention due to their antioxidant activities. Several studies have reported that MRPs possess in vitro reducing power, free radical scavenging capacity, and metal chelating ability [1,2,3,4,5]. MRPs are naturally found in food products such as coffee, beer, and baked goods, but can also be produced intentionally using proteins and carbohydrates as precursors [6,7]. Many studies have focused on evaluating the antioxidant properties of MRPs using standard in vitro assays. However, these indirect methods, which often do not involve oxidizable substrates, may not fully represent the antioxidant mechanisms relevant in real food systems [8]. This limitation is common to many antioxidant compounds [7]. As a result, there has been growing interest in investigating the effectiveness of MRPs in complex food systems such as bulk oils and oil-in-water (O/W) emulsions, where lipid oxidation leads to quality loss [7,9,10,11,12].

To date, several studies have highlighted the promising antioxidant potential of MRPs, particularly within model food systems. For instance, MRPs have been shown to protect omega-3 fatty acids such as EPA and DHA in krill oil during accelerated storage by slowing lipid oxidation [13]. Similarly, MRPs derived from soybean protein isolate and polysaccharides significantly improved the stability of citral, a volatile aroma compound, by offering enhanced protection during long-term storage, heat treatment, and simulated gastrointestinal digestion [14]. In oil-in-water emulsions, MRPs contribute to oxidative stability through multiple mechanisms, including the formation of a protective layer at the oil-water interface, scavenging of surface free radicals, and antioxidant activity within the aqueous phase [7,9,10]. More recently, MRPs produced from phosphatidylethanolamine and xylose were reported to exhibit high reactivity and strong antioxidant capacity, effectively improving the oxidative stability of flaxseed oil and reducing the formation of aldehydes under accelerated oxidation conditions [12]. Collectively, these findings reinforce the role of MRPs in enhancing oxidative stability in model systems and support their potential application as natural, clean-label antioxidants in food product development.

MRPs are typically produced by heating amino acids with reducing sugars in water or buffered solutions. However, the type of reaction medium can play a crucial role in determining both the reaction rate and the characteristics of the resulting MRPs. While most studies use water or buffer systems, only a few have explored alternative media such as alcohol, vinegar, or sugar syrup [15,16,17,18,19]. To date, the use of ozonized water as a reaction medium in the Maillard reaction has not been reported. Ozone is a strong oxidizing agent that is considered environmentally friendly and has been approved by the U.S. Food and Drug Administration (FDA) for use in food processing. It is commonly applied for sanitizing equipment, purifying water, and improving the quality and appearance of food products such as seafood and surimi, as well as mildly salted duck egg [20,21,22,23]. Due to its high oxidation potential, ozonized water may alter the chemical structure of amino acids and sugars before or during the Maillard reaction. These changes could influence the reactivity of the substrates, potentially accelerating or, in some cases, slowing down the reaction, depending on the extent and type of modification. Moreover, ozonized water decomposes into oxygen, leaves no harmful residue, and supports current trends toward clean-label and sustainable food processing.

The objective of this study was to investigate the potential of ozonized water as a novel reaction medium for the Maillard reaction between glycine and fructose. The antioxidant activities of the resulting MRPs were evaluated through in vitro assays and within an oil-in-water emulsion model, and the outcomes were compared to those obtained using a conventional phosphate buffer system. It was hypothesized that the use of ozonized water, due to its high oxidation potential, could influence the reaction kinetics and structural characteristics of MRPs, thereby influencing their antioxidant properties. This approach aimed to explore a cleaner, residue-free alternative for producing functional MRPs suitable for application as natural antioxidants in food systems.

## 2. Materials and Methods

### 2.1. Chemicals

Fructose, glycine, 2,2-diphenyl-1-picrylhydrazyl (DPPH), 2,2′-azino-bis(3-ethylbenzothiazoline-6-sulfonic acid) (ABTS), ethylenediaminetetraacetic acid (EDTA), potassium iodide (KI), hydrochloric acid (HCl), chloroform, acetic acid, trichloroacetic acid (TCA), thiobarbituric acid (TBA), 1,1,3,3-tetramethoxypropane, ferrous (II) sulfate heptahydrate (Fe(II)SO_4_.7H_2_O), Tween 20, and other analytical-grade chemicals were purchased from Sigma-Aldrich (St. Louis, MO, USA).

### 2.2. Formulation of a Model System for Fructose-Glycine Maillard Reaction

Ozonized water containing 50 ng ozone/mL was generated using an Ozoner^®^-020 ozone generator (ProTechSci Co., Chiang Mai, Thailand; maximum output 500 mg/h) and used within 30 min to minimize the loss of reactive species. The ozone concentration was controlled based on the calibrated output setting of the ozone generator under standardized operating conditions rather than direct chemical quantification, as aqueous ozone is highly unstable and decomposes rapidly [23]. The selected concentration (50 ng/mL, equivalent to 0.05 mg/L) was determined through preliminary optimization trials to provide sufficient oxidative influence while avoiding excessive degradation of reactants. Previous studies on aqueous ozone applications in food systems have reported effective dissolved ozone concentrations ranging from 0.15 to 36 mg/L for antimicrobial and quality effects, with treatment conditions being highly product-specific [24,25,26]. Moreover, regulatory approvals indicate that low dissolved ozone levels (e.g., up to ~0.4 mg/L) are considered safe for food processing applications [27,28]. This ozonized water initially had a pH of 7.38, but upon dissolving fructose and glycine, the pH increased to approximately 7.80. Therefore, a 0.2 M phosphate buffer at pH 7.80 was used as a control to match the reaction conditions. Fructose and glycine were each dissolved at a final concentration of 0.2 M in either ozonized water or phosphate buffer (total volume of 1 L in a screw-capped DURAN^®^ laboratory bottle). The mixtures were heated at 100 °C in a temperature-controlled water bath (W350, Memmert, Schwabach, Germany) for up to 120 min to initiate the Maillard reaction. Aliquots (10 mL) were collected at 0, 15, 30, 60, and 120 min, rapidly cooled in ice water, and immediately subjected to subsequent analyses.

### 2.3. Measurement of Intermediate Product and Browning Intensity

The intermediate product and browning intensity of the Maillard reaction were measured by the absorbance at 294 (A294) and 420 nm (A420), respectively, using a Shimadzu UV-2100 spectrophotometer (Shimadzu Scientific Instruments Inc., Columbia, MD, USA) [19]. Prior to analysis, appropriate dilutions with distilled water were made.

### 2.4. Determination of Color

Color of the sample was measured using a portable Hunterlab Miniscan/EX instrument (10° standard observers, illuminant D65, Hunter Assoc. Laboratory; Reston, VA, USA). The L* (lightness), a* (redness/greenness), and b* (yellowness/blueness) were reported.

### 2.5. Determination of pH and Electrical Conductivity

The pH was measured using a pH meter (Cyberscan 500, Singapore), while electrical conductivity was determined with a conductometer (WTW inoLab Cond 700, WTW, Weilheim, Germany) at room temperature (27–29 °C) [29].

### 2.6. Determination of Antioxidant Activities

The antioxidant activity of MRPs was evaluated using DPPH and ABTS radical scavenging assays, following the method of Limsuwanmanee et al. [1]. The results were expressed as percentage inhibition of radical activity.

For the DPPH assay, 0.3 mL of the sample was mixed with 1.5 mL of 0.10 mM DPPH solution prepared in methanol. The mixture was vigorously shaken and then incubated in the dark at room temperature for 30 min. Absorbance was measured at 517 nm using a spectrophotometer (Shimadzu, Columbia, MD, USA ).

In the ABTS assay, 20 μL of the sample was added to 2 mL of 7 mM ABTS^∙+^ solution. After incubation at room temperature for 6 min, the absorbance was recorded at 734 nm.

In both assays, distilled water was used in place of the sample to prepare the blank solution.

### 2.7. Oxidative Stability of Emulsion Model Systems Supplemented with MRPs

The emulsion model system was prepared following the method of Irankunda et al. [30] with slight modifications. An oil-in-water emulsion containing 5% (*w*/*w*) soybean oil (Oleen Co., Ltd., Bangkok, Thailand) was formulated. The aqueous phase comprised 94% (*w*/*w*) of the designated solvent, containing 50 µM ferrous sulfate as a prooxidant. Treatments included distilled water (control), 200 µM EDTA, and Maillard reaction products (MRPs) produced in ozonized water blended with MRPs from phosphate buffer at weight ratios of 75:25, 50:50, and 25:75. These blending ratios were selected to create a systematic compositional gradient, allowing evaluation of dilution effects as MRPs produced in ozonized water were progressively replaced by buffered MRPs in 25% increments. This design enables assessment of trend-dependent changes in oxidative stability across increasing proportions of buffered MRPs, bridging the transition from predominantly ozonized MRPs toward buffered MRPs. Tween 20 at 1% (*w*/*w*) was added as an emulsifier. The mixture was homogenized at 16,000 rpm for 3 min to form a stable emulsion.

The emulsions with different additives were transferred into screw-capped DURAN^®^ laboratory bottles, wrapped in aluminum foil, and stored in the dark at room temperature (27–29 °C) for 3 days. Lipid oxidation was assessed by measuring peroxide value (PV) and thiobarbituric acid reactive substances (TBARS) at Days 0 and 3. The rates of peroxide and TBARS formation were calculated relative to the initial values at Day 0. Zeta-potential was also measured to evaluate emulsion stability and potential signs of destabilization.

#### 2.7.1. Determination of PV

The sample (1 g) was treated with 25 mL of a chloroform–acetic acid mixture, 2:3. The mixture was shaken vigorously, followed by the addition of 1 mL of saturated KI. The mixture was kept in the dark for 5 min, and 75 mL of distilled water was added subsequently. A 0.5 mL starch solution (1%, *w*/*v*) was added as an indicator. The PV was determined by titrating the iodine liberated from potassium iodide with a standardized 0.01 N sodium thiosulfate solution. The PV was expressed as milliequivalents of free iodine/kg of sample [31].

#### 2.7.2. Determination of TBARS

TBARS were determined following the method of Wongnen et al. [23]. A 0.5 g sample was mixed with 2.5 mL of TBARS reagent containing 0.375% TBA, 15% TCA, and 0.25 N HCl. The mixture was homogenized at 13,500 rpm for 30 s, then incubated in a water bath at 95 °C for 10 min. After cooling under running tap water, the mixture was centrifuged at 3600× *g* for 20 min at 25 °C. The absorbance of the supernatant was measured at 532 nm. A standard curve was prepared using 1,1,3,3-tetramethoxypropane in the range of 0–10 ppm, and TBARS values were expressed as milligrams of malondialdehyde (MDA) equivalents per kilogram of sample.

#### 2.7.3. Determination of Zeta-Potential

The zeta-potential of the samples was measured according to the method of Chaijan et al. [32] using a Zetasizer Nano-ZS90 (Malvern Instruments Ltd., Malvern, Worcestershire, UK).

### 2.8. Statistical Analysis

Data were subjected to the analysis of variance (ANOVA). Comparison of means was carried out by Duncan’s multiple-range test to indicate significant differences (*p* < 0.05). The Statistical Package for Social Science (SPSS 23.0 for Windows, SPSS Inc., Chicago, IL, USA) was used.

## 3. Results and Discussion

### 3.1. Effect of Ozonized Water vs. Buffer on Maillard Reaction Progression During Heating

#### 3.1.1. Visual Appearance and Color Observation

**Figure 1:**
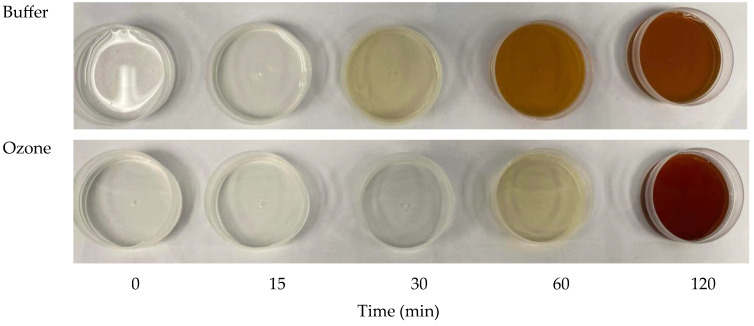
Changes in appearance of fructose-glycine Maillard reaction models prepared in buffer and ozonized water.

The visual appearance of the fructose-glycine model system heated at 100 °C at various time points (0–120 min) demonstrates the development of MRPs, with notable differences between samples prepared in buffer and those in ozonized water (Figure 1). According to Koubaa et al. [33], the Maillard reaction progresses through three stages. In the initial stage, sugar-amine condensation and Amadori rearrangement occur, producing colorless intermediates with minimal ultraviolet (UV) absorption. The intermediate stage is characterized by the degradation of sugars and amino acids, leading to the formation of yellow compounds with distinct UV absorption. Finally, in the advanced stage, complex reactions generate heterocyclic N-containing compounds, resulting in the development of characteristic brown pigments.

During the first 30 min, there was minimal browning in both systems. At the early stages (0–15 min), both systems remained clear and colorless, indicating the absence of significant Maillard intermediates. By 30 min, a slight yellow hue appeared in the buffer system, while the ozonized water sample remained nearly colorless. This suggests that ozone pretreatment delays the initial formation of color-producing intermediates, likely by oxidizing available amino groups (e.g., glycine) [34] or by modifying the reactivity of reducing sugars [35]. At 60 min, the buffer system exhibited a distinct orange-brown color, probably due to the formation of furfurals, reductones, and low-molecular-weight melanoidins [36]. In contrast, the ozonized water sample developed only a light-yellow tint, further supporting the inhibitory or delaying effect of ozone on Maillard progression. At 120 min, both systems showed significant browning. The buffer system turned dark amber, typical of advanced Maillard reactions. Interestingly, the ozonized water system exhibited an even deeper reddish-brown color. This may reflect a shift in the reaction pathway, where ozone-induced oxidative changes promote the formation of MRPs with higher conjugation and nitrogen content, possibly yielding chromophores with greater redness and darker tonality. Overall, heating the fructose-glycine model system in ozonized water delays early Maillard reaction stages compared to the buffer. However, prolonged heating leads to even more intense browning, likely due to ozone-modified precursors favoring alternative MRP pathways. These findings suggest that ozone pretreatment alters the kinetics and nature of Maillard product formation rather than merely suppressing it.

#### 3.1.2. Formation of Intermediate Browning and Brown Pigment

The Maillard reaction begins with the interaction between the carbonyl group of a reducing sugar and the amino group of an amino acid, leading to the formation of colorless Amadori compounds that exhibit UV absorption, typically measured at 294 nm (A294). These intermediates gradually transform into nitrogen-containing brown pigments known as melanoidins [19,36]. The accumulation of melanoidins is commonly assessed by monitoring absorbance at 420 nm (A420) [37].

**Figure 2:**
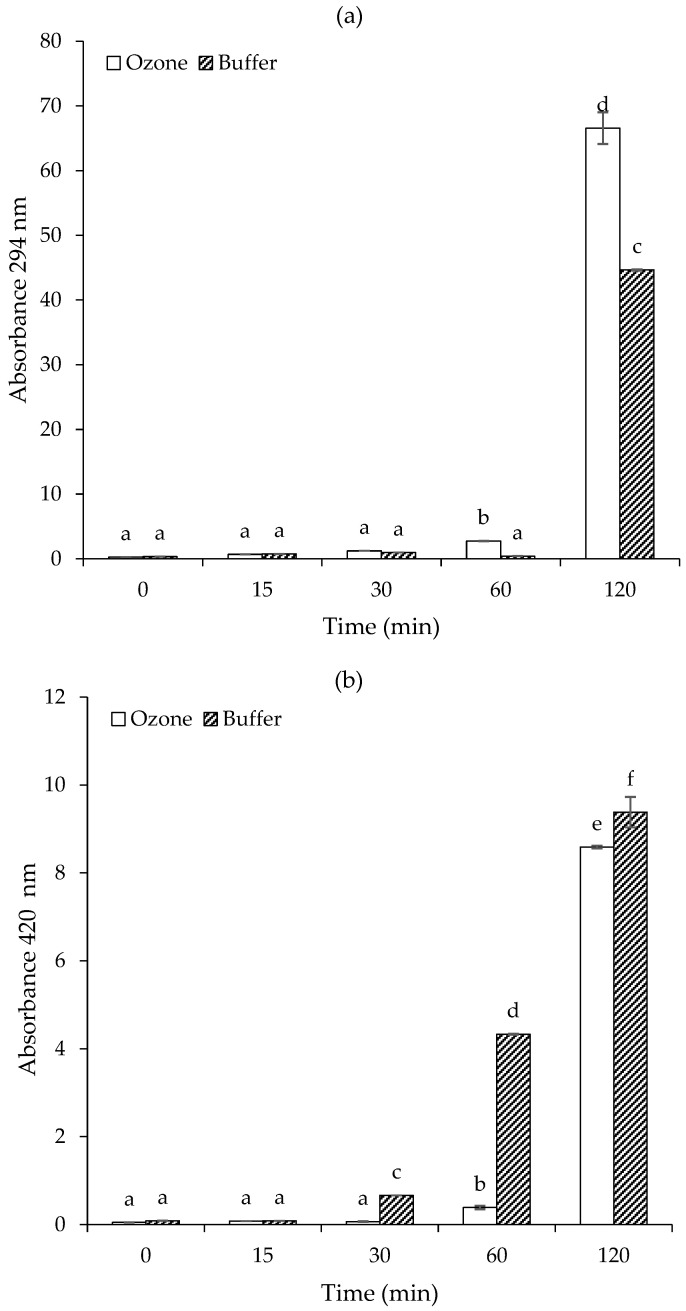
Changes in absorbance at 294 nm (**a**) and 420 nm (**b**) of fructose-glycine Maillard reaction models prepared in buffer and ozonized water. Bars represent standard deviation from triplicate determinations. Different letters indicate significant differences among samples (*p* < 0.05).

The intermediate browning measured by A294 of the fructose-glycine model system in ozonized water versus buffer is shown in Figure 2a. A294 is commonly used to monitor the accumulation of Maillard reaction intermediates, such as reductones and carbonyl-amine adducts, which precede the formation of brown melanoidins [19]. During the initial 30 min of heating, A294 values for both ozone- and buffer-treated samples remained very low and statistically similar (*p* > 0.05), indicating minimal formation of UV-absorbing intermediates. This matches the visual observation of clear or faintly yellow solutions and suggests that the early stage of the Maillard reaction was slow under both conditions. At 60 min, a significant increase in A294 was observed in the ozone-treated sample (*p* < 0.05), while the buffer-treated sample still showed low absorbance. This indicates that ozonized water accelerated the formation of intermediate Maillard products at this stage, likely due to ozone-induced oxidation producing more reactive carbonyls or facilitating new reaction pathways. Given ozone’s high oxidizing potential (E° = 2.07 V, which is higher than that of hydrogen peroxide, chlorine gas, or molecular oxygen) [26], it is also plausible that some reactive carbonyl intermediates and antioxidant-capable primary products were partially consumed, delaying the apparent accumulation of early-stage intermediates rather than completely inhibiting their formation. After 120 min, the ozonized system showed a markedly higher A294 value compared to the buffer system (*p* < 0.05). This confirms that ozone pretreatment significantly enhanced the accumulation of intermediate MRPs. The elevated A294 suggests intensified formation of reductones, Strecker aldehydes, and related species, which are precursors to final brown pigments [19]. These observations align with previous reports showing that oxidative modification of Maillard intermediates can redirect reaction pathways and influence intermediate accumulation, for example, via reactive α-dicarbonyl trapping or oxidation [38,39]. The results reveal that while ozonized water initially delays browning visually, it strongly promotes the formation of intermediate Maillard products as evidenced by higher A294 values at later heating stages. This suggests that ozone alters the chemical environment, possibly through oxidative modification of reactants, redirecting the Maillard pathway toward increased intermediate and eventual pigment production.

A420 reflects the formation of high-molecular-weight brown pigments, known as melanoidins, generated in the final stages of the Maillard reaction [37]. At 0–15 min, both ozone- and buffer-treated samples showed negligible A420 values, with no statistical difference (*p* > 0.05; Figure 2b). This indicates that melanoidin formation had not yet occurred, consistent with a slow initiation of the Maillard reaction under short-term heating. At 30 min, the buffer system exhibited a significant increase in A420 (*p* < 0.05), while the ozonized sample remained near baseline (*p* > 0.05). By 60 min, the buffer system reached an A420 of about 4.5, significantly higher than the ozone group (*p* < 0.05). These findings suggest that ozone pretreatment initially inhibits or delays the formation of final browning products, likely by modifying the availability or reactivity of key Maillard intermediates (e.g., glycine or fructose-derived carbonyls) [34,35]. This delay may result from partial consumption or oxidative transformation of early-stage intermediates by ozone, consistent with its strong oxidizing nature [26]. At 120 min, both systems showed intense browning, with A420 values reaching ~9.0 for the ozone sample and ~10.0 for the buffer sample. Although the buffer still yielded a slightly higher absorbance (*p* < 0.05), the ozone-treated system showed a strong compensatory increase in melanoidin formation, indicating that browning was not permanently suppressed but rather delayed. These results demonstrate that ozonized water delays the onset of final Maillard browning compared to buffer, as reflected by lower A420 up to 60 min. However, extended heating (120 min) allowed the ozonized system to develop comparable levels of melanoidins. This supports the idea that ozone modulates the Maillard pathway kinetics, delaying pigment formation in early stages, but ultimately leading to a rich profile of browning products with prolonged thermal exposure.

#### 3.1.3. Changes in Instrumental Color Parameters

**Figure 3:**
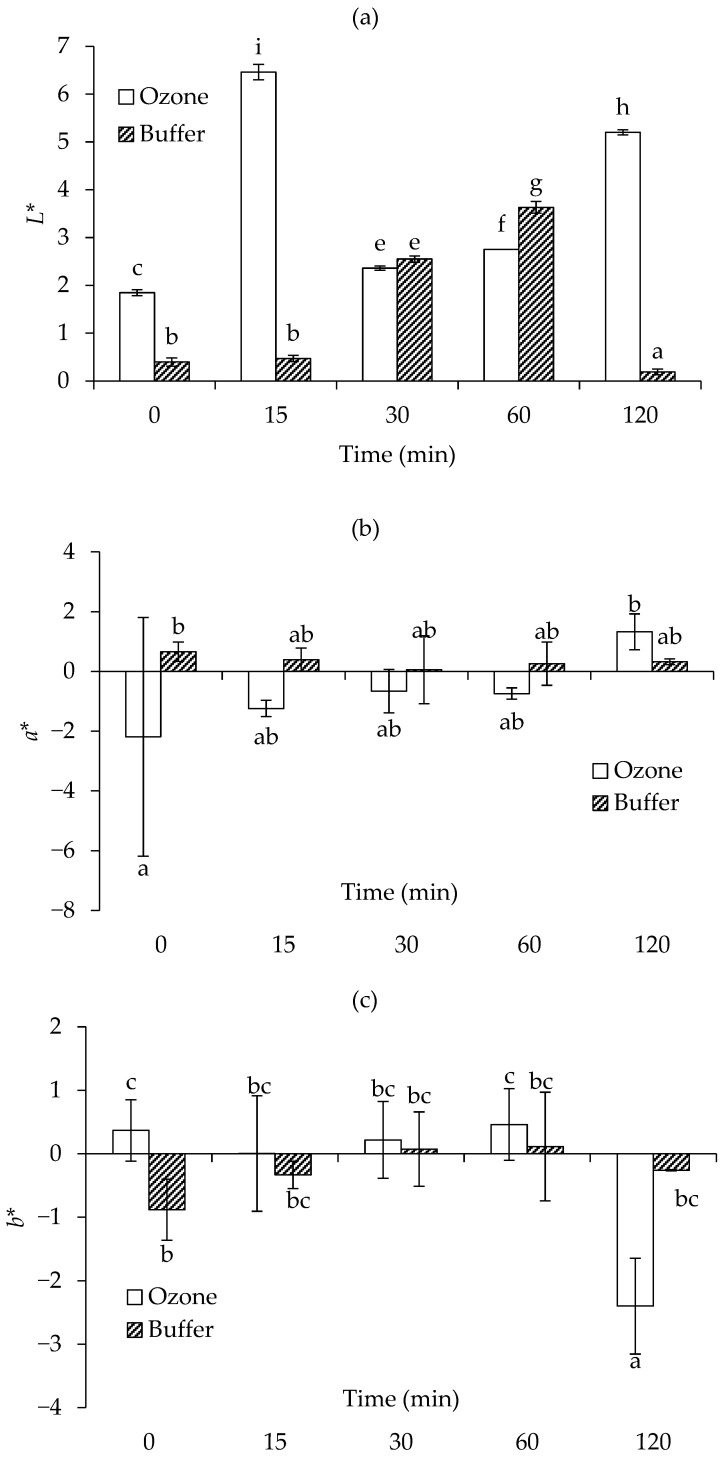
Changes in L* (**a**), a* (**b**), and b* (**c**) values of fructose-glycine Maillard reaction models prepared in buffer and ozonized water. Bars represent standard deviation from triplicate determinations. Different letters indicate significant differences among samples (*p* < 0.05).

Figure 3a–c illustrates the temporal evolution of L*, a*, and b* values in fructose-glycine Maillard-reaction model systems prepared in ozonized water or a phosphate buffer. At 0 min, both systems had fairly low L* because the starting mixture contains unreacted sugar and amino acid, although the ozone sample is marginally lighter. Heating for 15 min produced a marked divergence where the ozone treatment exhibits a sharp rise in L*, evidencing a pronounced lightening, whereas the buffer sample remains virtually unchanged. As heating continued, L* in the ozone system declined slightly at 30 min, then increased steadily and remained significantly higher (*p* < 0.05) than the initial value up to 120 min, indicating sustained suppression of browning. In contrast, the buffer system underwent progressive darkening; its L* dropped modestly at 30–60 min and reached a minimum near zero at 120 min, reflecting extensive formation of melanoidin pigments (Figure 3a). Changes in a* values (Figure 3b) were minor in both treatments, oscillating around zero without a consistent trend, implying that red- or green-hued chromophores contributed little to overall color development. The b* coordinate (Figure 3c) showed modest treatment-specific behavior: in ozone samples, it remained statistically unchanged through 60 min before falling at 120 min (*p* < 0.05), whereas in buffer samples it rose slightly between 0 and 15 min and then plateaued. The eventual shift in b* toward negative values suggested degradation or conversion of yellow intermediates to darker, bluish-tinted products as the Maillard reaction proceeded. In general, tanning reactions progress through distinct color transitions, typically shifting from yellow to red and eventually to dark brown. The rate and extent of color formation are influenced by factors such as the type of reacting compounds, their molar ratios, reaction duration, and pH-temperature conditions [40].

Collectively, these metrics demonstrated that ozonized water markedly retarded browning, preserving lightness and limiting chromophore formation, while the buffered system promoted classic Maillard-derived darkening.

#### 3.1.4. Changes in pH and Electrical Conductivity

**Figure 4:**
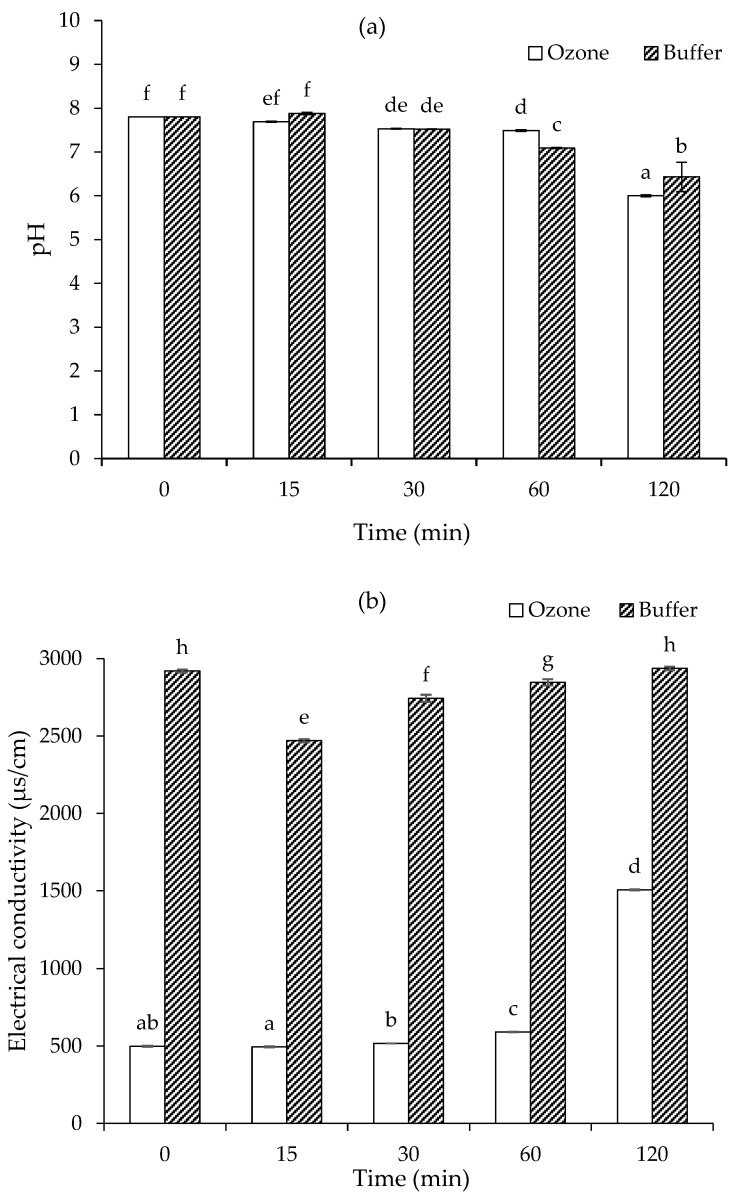
Changes in pH (**a**) and electrical conductivity (**b**) of fructose-glycine Maillard reaction models prepared in buffer and ozonized water. Bars represent standard deviation from triplicate determinations. Different letters indicate significant differences among samples (*p* < 0.05).

Figure 4a illustrates the evolution of pH in fructose-glycine model systems prepared in either phosphate buffer or ozonized water during heating. In both systems, pH remained stable during the first 15 min, followed by a gradual decline, reaching the lowest values at 120 min (*p* < 0.05). At this point, the pH of the ozonized sample dropped significantly to 6.00, compared to 6.43 in the buffered system. The ozonized system exhibited a sharp decrease in pH between 60 and 120 min, coinciding with a rapid progression of the Maillard reaction, evidenced by the sudden development of a dark-brown color (Figure 1). In contrast, the buffered system showed a more gradual and continuous browning progression, accompanied by a slower pH decline, likely due to the buffering capacity of the phosphate buffer. It should be noted that the use of phosphate buffer inherently stabilizes pH and may partially mask reaction-induced pH changes; therefore, comparisons between buffered and ozonized systems are interpreted in a relative rather than absolute manner. The observed pH changes in the ozonized system arise from a combination of ozone decomposition and the formation of acidic Maillard intermediates and products, rather than solely from ozone consumption. The overall reduction in pH in both systems is attributed to the accumulation of organic acids formed during the Maillard reaction, consistent with previous findings [19,41].

Figure 4b illustrates the changes in electrical conductivity of fructose-glycine Maillard reaction models prepared in buffer and ozonized water during heating. At all time points, the buffered system exhibited significantly higher conductivity than the ozonized system (*p* < 0.05), primarily due to the presence of phosphate salts contributing additional mobile ions. Accordingly, the buffered system does not represent an inert control for conductivity measurements, and the influence of buffer-derived ions must be considered when interpreting these results. In the buffered sample, conductivity decreased slightly during the initial 15 min of heating (*p* < 0.05), likely due to early-stage complexation between fructose and glycine forming neutral intermediates such as Schiff bases or Amadori products, which temporarily reduce free ion concentration [42]. Additionally, phosphate buffer components may undergo thermal re-equilibration (e.g., shifts between H_2_PO_4_^−^ and HPO_4_^2−^ species), transiently affecting ion mobility. Following this phase, conductivity in the buffered system gradually increased, returning to its initial value by 120 min as the Maillard reaction progressed. In contrast, the ozonized system showed minimal change in conductivity during the first 30 min, followed by a modest increase at 60 min and a sharp rise by 120 min, indicating delayed but rapid Maillard reaction progression. The increasing conductivity in both systems over time is attributed to the accumulation of ionic and polar MRPs, particularly low-molecular-weight organic acids (e.g., formic, acetic, pyruvic), charged intermediates, and degradation products of sugars and amino acids [43]. Some of these products may interact with free ions, either through transient binding or incorporation into reaction intermediates, which could reduce ion mobility and contribute to the observed conductivity patterns. The concurrent decrease in pH enhances ionization of weak acids, further contributing to conductivity [44]. Importantly, conductivity here is used as an indirect physicochemical indicator of Maillard reaction progression rather than as direct evidence for ion chelation or other specific interactions. No structural or quantitative evidence was obtained for metal binding, and any such interpretations remain speculative. Overall, the observed trends highlight conductivity as a useful comparative indicator of changes in the reaction medium and differences in reaction dynamics between buffered and ozonized systems, without implying direct causation or chelation. Future studies incorporating pH-adjusted pure water systems as additional controls would help to better isolate buffer-related effects from intrinsic reaction-induced changes in conductivity.

It should be noted that in this study we monitored Maillard reaction progression using a combination of indirect indicators, including absorbance at A294 and A420, visual observation of color, instrument-based color measurements, pH, and electrical conductivity. While these measures provide useful comparative information, they do not allow full structural characterization of the intermediates or final Maillard products. Future studies using chromatographic and spectrometric analyses are needed to identify and quantify specific reaction products, providing a more complete mechanistic understanding.

### 3.2. In Vitro Antioxidant Activities of MRPs

The antioxidant activity of MRPs arises from both the accumulation of UV-absorbing intermediates and the formation of highly active final products, such as melanoidins [8,45]. These intermediates and brown pigments act as effective hydrogen donors, thereby exhibiting antiradical activity [19,41]. Melanoidins can inhibit lipid peroxidation by suppressing the formation of both primary and secondary oxidation products. Their metal-chelating capacity, as demonstrated in melanoidins from barley coffee, dark beer, and roasted coffee, further limits pro-oxidative reactions during digestion, contributing to potential health benefits [46].

**Figure 5:**
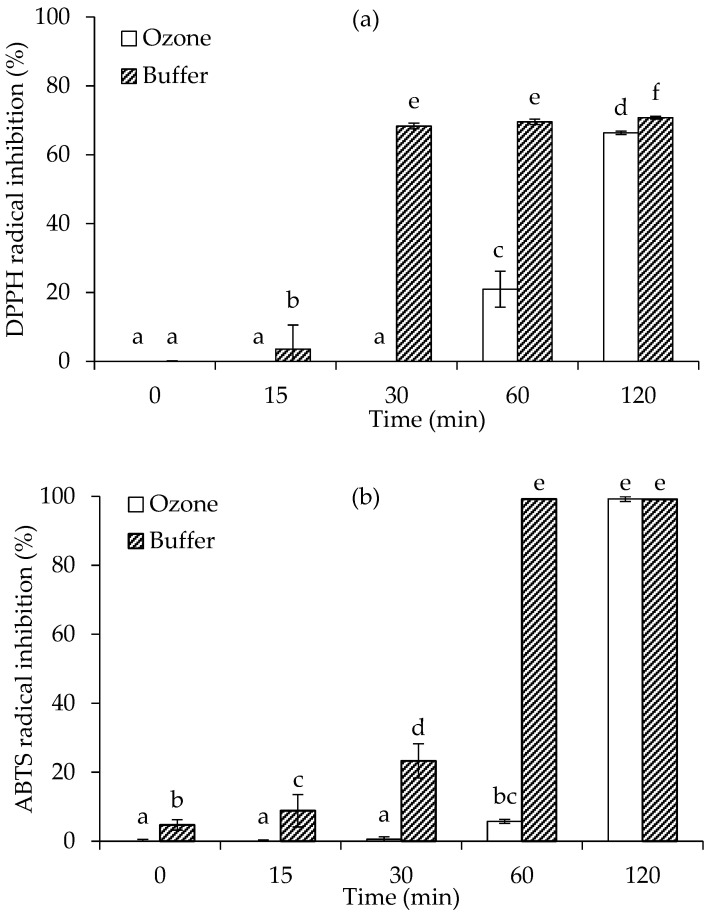
Changes in DPPH radical scavenging activity (**a**) and ABTS radical scavenging activity (**b**) of fructose-glycine Maillard reaction models prepared in buffer and ozonized water. Bars represent standard deviation from triplicate determinations. Different letters indicate significant differences among samples (*p* < 0.05).

Figure 5a illustrates the changes in DPPH radical scavenging activity of fructose-glycine Maillard reaction models prepared in buffer and ozonized water over time. At 0 min, both treatments exhibited minimal inhibition, with no significant difference (*p* > 0.05). By 15 min, the buffer treatment showed a slight but significantly higher DPPH inhibition compared to the ozone sample (*p* < 0.05). A clear divergence emerged at 30 min, where the buffer system reached approximately 70% inhibition, while the ozone-treated sample remained low. This trend persisted at 60 min, with the buffer maintaining high scavenging activity and the ozone treatment showing only a modest increase. By 120 min, both treatments achieved similarly high DPPH radical inhibition, indicating that although antioxidant activity developed more slowly in the ozonized water system, the final outcome approached that of the buffer system with prolonged heating.

Figure 5b shows the ABTS radical scavenging activity of the same model systems. At the beginning (0 min), both buffer and ozone treatments demonstrated negligible inhibition. Between 0 and 15 min, the buffer system exhibited a slight but significantly higher ABTS inhibition, whereas the ozone treatment remained very low (*p* < 0.05). At 30 min, this difference became more evident: the buffer reached ~25% inhibition, while the ozone sample remained largely inactive (*p* < 0.05). A substantial increase occurred at 60 min, with the buffer system achieving nearly 100% ABTS inhibition. In contrast, the ozone treatment showed only a slight increase at this stage (*p* < 0.05). However, by 120 min, both treatments attained comparable, near-complete ABTS scavenging activity, confirming that antioxidant potential in the ozone system eventually caught up with the buffer treatment after extended heating. When compared to DPPH scavenging activity (Figure 5a), a similar trend of delayed antioxidant development in the ozonized water system was observed for both assays. However, the ABTS assay generally demonstrated a more rapid and complete increase in antioxidant activity, achieving near-maximal inhibition by 60 min in the buffer and 120 min in the ozone treatment, whereas the DPPH assay reached lower maximum inhibition levels (approximately 70%) even at 120 min (Figure 5a,b). This suggested that Maillard reaction products were either more effective in scavenging ABTS radicals than DPPH radicals or that the kinetics of interaction varied between the two radical systems, with ABTS being more responsive to the polar and ionic compounds formed [47]. Additionally, the increase in conductivity observed in Figure 4b reflected the accumulation of ionic and polar Maillard intermediates, which appeared to correspond more closely with ABTS radical scavenging activity. At 120 min, the ozone system retained a higher proportion of intermediate products, while the buffer system had progressed to form more final-stage brown pigments. Since antioxidant activity could arise from both intermediate and final Maillard reaction products, as evidenced by the activity observed during both early and late stages of heating, the nature and balance of these compounds played a key role in determining the overall radical scavenging performance [48]. The combination of Maillard reaction products derived from both buffer- and ozone-based preparations may serve as a strategic approach to enhance antioxidant activity, owing to the presence of a broader spectrum of antioxidant compounds with complementary properties.

### 3.3. Antioxidant Activity of MRPs in Emulsion Model System

#### 3.3.1. Rate of Peroxide Formation

**Figure 6:**
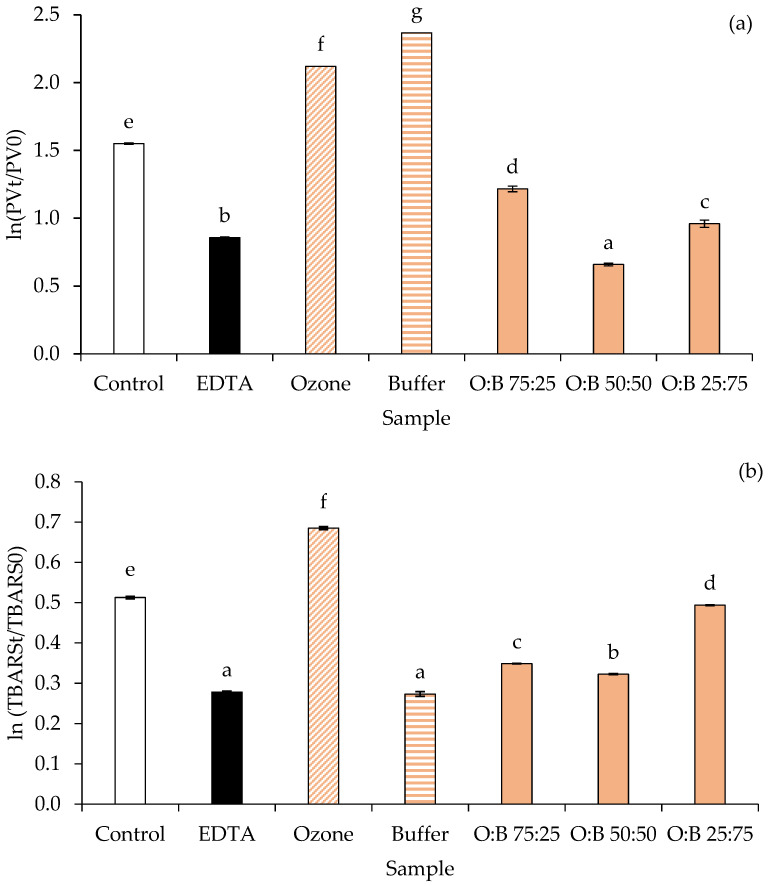
Rate of peroxide formation (**a**) and rate of TBARS formation (**b**) in emulsion treated with different additives. Bars represent standard deviation from triplicate determinations. Different letters indicate significant differences among samples (*p* < 0.05). PV_t_ = Peroxide value during storage at room temperature (27–29 °C) at Day 3. PV_0_ = Peroxide value of fresh emulsion at Day 0. TBARS_t_ = TBARS content during storage at room temperature at Day 3. TBARS_0_ = TBARS content of fresh emulsion at Day 0. Ozone = MRPs produced in ozonized water. Buffer = MRPs produced in buffer. O:B = MRPs produced in ozonized water blended with MRPs produced in buffer at specified ratios.

Figure 6a illustrates the rate of peroxide formation, expressed as ln(PV_t_/PV_0_), in an emulsion treated with various additives, with higher values indicating a greater rate of peroxide formation and thus less oxidative stability. The control sample exhibited a relatively high rate of peroxide formation, indicating inherent susceptibility to oxidation. EDTA, a known chelating agent, significantly reduced the rate of peroxide formation compared to the control, demonstrating its effectiveness in inhibiting lipid oxidation [49]. Conversely, samples treated with MRPs generated in ozone and buffer systems alone exhibited significantly higher rates of peroxide formation compared to the control and EDTA. The highest rate was observed in the buffer-derived MRPs, suggesting that when applied individually, these MRPs promoted lipid oxidation rather than inhibiting it under the experimental conditions. This occurred despite their demonstrated free radical scavenging activity in vitro (Figure 5a,b), highlighting a potential discrepancy between antioxidant behavior in chemical assays and real food systems. However, when MRPs produced in ozonized water (O) were blended with MRPs produced in buffer (B) at various ratios (O:B 75:25, 50:50, and 25:75), the rate of peroxide formation significantly decreased compared to the individual ozone and buffer MRP treatments. Specifically, the O:B 75:25 blend showed the lowest rate, comparable to or even slightly lower than the control, indicating a synergistic or antagonistic effect when blended that improved oxidative stability. The decreasing trend in peroxide formation as the proportion of buffer-produced MRPs increased in the blends (from O:B 75:25 to 25:75) suggests that the presence of buffer-produced MRPs, when combined with ozone-produced MRPs, might contribute to mitigating the pro-oxidant effect observed when they are used alone.

#### 3.3.2. Rate of TBARS Formation

Figure 6b presents the rate of TBARS formation, expressed as ln(TBARS_t_/TBARS_0_), in an emulsion treated with various additives, where higher values indicate a greater accumulation of secondary lipid oxidation products and thus lower oxidative stability. The control sample exhibited a moderate rate of TBARS formation. EDTA significantly reduced TBARS formation compared to the control, confirming its antioxidant efficacy by chelating metal ions that catalyze lipid oxidation [49]. In contrast, MRPs produced in ozone showed the highest rate of TBARS formation, indicating a strong pro-oxidant effect. Interestingly, MRPs produced in buffer showed a lower rate of TBARS formation than the control and ozone, suggesting some antioxidant capacity or a less pronounced pro-oxidant effect compared to ozone-produced MRPs when used alone. When MRPs produced in ozonized water were blended with MRPs produced in buffer (O:B 75:25, 50:50, and 25:75), the TBARS formation rates varied. The O:B 75:25 and O:B 50:50 blends showed TBARS formation rates higher than buffer-alone but lower than ozone-alone, indicating that blending could modulate the pro-oxidant effect of ozone-produced MRPs. Notably, the O:B 25:75 blend exhibited a significantly higher rate of TBARS formation compared to the other blends and even the control, suggesting that a higher proportion of buffer-produced MRPs in combination with ozone-produced MRPs did not necessarily lead to improved stability in this specific ratio, potentially indicating complex interactions.

In oil-in-water emulsions, oxidative stability is improved by MRPs through multiple mechanisms. These include the development of a protective interfacial layer, scavenging of free radicals at the oil-water boundary, and antioxidant action within the aqueous phase [7,9,10]. Thus, blending ozone- and buffer-derived MRPs yields a spectrum of hydrophilic and hydrophobic antioxidants that can act in the aqueous, interfacial, and lipid domains of oil-in-water emulsions. A 50:50 O:B mixture, for instance, suppressed both primary and secondary lipid-oxidation products and thus could be incorporated into emulsified foods to enhance oxidative stability. However, detailed studies in real food matrices are still needed, as antioxidant behavior observed in vitro does not always translate directly to complex systems containing diverse ingredients.

It is highlighted that MRPs can exhibit dual redox behavior depending on their concentration, the reaction medium, and the presence of transition metals [50,51]. A study on model glucose-lysine and fructose-lysine systems demonstrated that different MRPs generated under varied synthesis conditions exhibited antioxidant, pro-oxidant, or inactive behavior when tested in a lipid system containing copper ions [52]. Thus, at lower concentrations or in systems lacking catalytic metals, MRPs primarily act as radical scavengers, whereas at higher concentrations or in the presence of metals, pro-oxidant effects may predominate. This behavior may explain why MRPs produced in buffer or ozonized water alone occasionally promoted lipid oxidation in the emulsion assays (Figure 6), despite demonstrating clear antioxidant activity in DPPH and ABTS assays (Figure 5).

When MRPs from ozonized water (O) and buffer (B) were combined, antagonistic interactions between reactive species could occur, potentially neutralizing some pro-oxidant effects. Additionally, certain MRPs may preferentially scavenge reactive intermediates generated by other components. These interactions likely account for the improved inhibition of peroxide and TBARS formation observed in blends such as O:B 50:50 or 75:25 compared to the individual MRPs.

Overall, combining MRPs from different reaction media provides a broader spectrum of antioxidant compounds with complementary properties, enhancing oxidative stability in emulsion systems. While these observations offer mechanistic insights into the dual antioxidant and pro-oxidant roles of MRPs, detailed structural characterization and studies in real food systems are needed to fully elucidate their activity.

#### 3.3.3. Relationship Between PV and TBARS Formation

PV and TBARS are both critical indicators of lipid oxidation, but they measure different stages of the process. PV quantifies primary oxidation products, specifically hydroperoxides, which are the initial compounds formed when lipids react with oxygen. TBARS, on the other hand, measures secondary oxidation products, primarily MDA and other aldehydes, which are formed from the breakdown of these primary hydroperoxides [53].

Figure 6a,b illustrate a clear relationship between PV and TBARS formation. Treatments that exhibited high PV in Figure 6a generally also showed elevated TBARS levels in Figure 6b. For example, the ozone treatment consistently produced very high PV and TBARS values, indicating that MRPs generated in ozonized water exerted a strong pro-oxidant effect, accelerating both the initial formation of lipid hydroperoxides and their subsequent degradation into secondary oxidation products. The buffer treatment alone also demonstrated a pro-oxidant effect; however, the extent differed between the two assays, with the buffer treatment showing the highest PV, whereas the ozone treatment produced the highest TBARS levels. This pattern suggests that buffer-derived MRPs efficiently initiate lipid oxidation but may generate TBARS-reactive compounds more slowly or through different secondary pathways compared to ozone-derived MRPs. In contrast, treatments that effectively suppressed PV formation, such as EDTA, also markedly reduced TBARS levels, confirming its broad-spectrum antioxidant activity. A notable finding was observed for the O:B blends. While the individual ozone and buffer MRPs both promoted lipid oxidation, blending them at specific ratios, particularly O:B 75:25, significantly reduced both PV and TBARS compared with the individual treatments. This indicates a potential synergistic or antagonistic interaction that limits hydroperoxide formation and slows their subsequent conversion into TBARS-reactive compounds, thereby enhancing overall emulsion stability.

The consistent trends between PV and TBARS across most treatments highlight that these assays together provide a comprehensive understanding of lipid oxidation, from the generation of primary hydroperoxides to the formation of secondary products. Furthermore, the findings underscore the complex and concentration-dependent effects of different MRPs and their blends on the oxidative stability of emulsions.

### 3.4. Emulsion Stability as Measured by Surface Charge

The charge of MRPs is multifaceted and dynamic, not confined to a single positive, negative, or neutral state; rather, it typically involves a mixture of all three, heavily influenced by the specific chemical structure of the diverse compounds formed and the pH of the surrounding environment. Starting from reducing sugars and amino acids, the complex series of Maillard reactions yields a wide spectrum of products, from early-stage Amadori compounds to intermediate dicarbonyls, and finally, complex polymeric melanoidins. These varied products possess different ionizable functional groups, such as amino groups (which can be protonated to a positive charge at acidic pH) and carboxyl groups (which can be deprotonated to a negative charge at alkaline pH) [54,55]. As the reaction often generates organic acids, the resulting pH changes can further alter the protonation states of these groups [19,41]. Consequently, while melanoidins, for example, often exhibit amphoteric properties due to both acidic and basic groups, their net charge at neutral to alkaline pH is commonly negative due to the prevalence of carboxylic acid functionalities, ultimately making the charge of the overall MRP mixture highly dependent on the particular conditions and the specific compounds present [56,57].

Table 1 presents the zeta-potential values of emulsion samples treated with various additives over a 3-day storage period, providing insights into their colloidal stability. At day 0, the control emulsion had a moderate negative zeta-potential. EDTA and the O:B blends (especially O:B 50:50 and 25:75) showed significantly less negative zeta-potential values compared to the control, ozone, and buffer-alone treatments, suggesting a potential reduction in electrostatic repulsion, which could generally lead to less stable emulsions [58]. Over 3 days of storage, all samples experienced a decrease in the absolute magnitude of their negative zeta-potential, indicating a reduction in surface charge and thus a decrease in emulsion stability, consistent with the expected destabilization over time. The greatest decrease in absolute zeta-potential for a single treatment was observed in the control, buffer, and O:B 75:25, suggesting relatively lower stability over time. Interestingly, while the ozone and buffer treatments individually led to higher PV and TBARS formation (as seen in Figure 6a,b), indicating lower oxidative stability, their initial zeta-potentials were more negative than the O:B blends, suggesting better initial electrostatic repulsion. However, the O:B blends, particularly O:B 50:50 and 25:75, maintained a relatively stable (though less negative) zeta-potential over the 3 days compared to the more pronounced shift in the control and buffer-alone treatments, implying that while they started with lower charge, they might resist further changes in charge more effectively. This observation highlights a potential disconnect between oxidative stability (PV and TBARS) and colloidal stability (zeta-potential); a system that effectively prevents lipid oxidation might not necessarily maintain optimal electrostatic repulsion, and vice versa, depending on the complex interactions of the MRPs with the emulsion components.

## 4. Conclusions

This study demonstrates that ozonized water is an effective medium for generating fructose–glycine MRPs with distinct physicochemical and functional properties compared to those produced in phosphate buffer. Ozone treatment delayed early browning but ultimately promoted the formation of highly conjugated, reddish-brown MRPs, accompanied by greater pH reduction and conductivity. Antioxidant activity in the ozone system developed more slowly yet reached similar levels to the buffer system after prolonged heating. In oil-in-water emulsions, individual MRPs acted as pro-oxidants, whereas blending ozone- and buffer-derived MRPs, particularly at 75:25 and 50:50 ratios, significantly suppressed lipid oxidation and enhanced charge stability. Thus, blending MRPs from different reaction media offers a promising strategy for improving antioxidant performance in multi-phase food systems. Future work should focus on characterizing the specific compounds responsible for antioxidant and pro-oxidant effects and validating the functionality of MRPs blends in real food matrices to support their application in clean-label emulsified products.

## Figures and Tables

**Table 1:** Zeta-potential (mV) of emulsion treated with different additives during storage in the dark at room temperature (27–29 °C) for 3 days.

Sample	Storage Time (Day)	
	0	3
Control	−14.60 ± 1.21 aA	−8.96 ± 0.68 aB
EDTA	−7.28 ± 0.18 dA	−5.56 ± 0.07 dB
Ozone	−10.73 ± 0.35 cA	−9.38 ± 0.06 aB
Buffer	−13.27 ± 0.06 bA	−7.19 ± 0.65 bB
O:B 75:25	−7.69 ± 0.43 dA	−6.23 ± 0.33 cB
O:B 50:50	−6.09 ± 0.39 eA	−4.72 ± 0.05 eB
O:B 25:75	−5.71 ± 0.08 eA	−4.68 ± 0.14 eB

Values are expressed as mean ± standard deviation from triplicate determinations. Lowercase letters (a–e) within the same column and uppercase letters (A, B) within the same row indicate significant differences (*p* < 0.05). Ozone = MRPs produced in ozonized water; Buffer = MRPs produced in buffer; O:B = MRPs produced in ozonized water blended with MRPs produced in buffer at the specified ratios.

## Data Availability

The original contributions presented in this study are included in the article. Further inquiries can be directed to the corresponding author.
